# Development of a reverse genetics system for Getah virus and characterization of rescued strains

**DOI:** 10.1186/s13567-025-01515-x

**Published:** 2025-04-12

**Authors:** Rongxuan Cai, Qi He, Qing Wang, Lan Tian, Zhe Chen, XiaoFeng Wu, Jiumeng Sun, Ying Shao, Xiangjun Song, Kezong Qi, Jian Tu, Zhenyu Wang

**Affiliations:** 1 Anhui Province Key Laboratory of Veterinary Pathobiology and Disease Control, College of Veterinary Medicine, Anhui Agricultural University, Hefei, 230036 China; 2 Anhui Province Engineering Laboratory for Animal Food Quality and Biosafety, College of Veterinary Medicine, Anhui Agricultural University, Hefei, 230036 China; 3 Joint Research Center for Food Nutrition and Health of IHM, Anhui Agricultural University, Hefei, 230036 China

**Keywords:** Getah virus, reverse genetics system, infectious clone, reporter virus

## Abstract

Getah virus (GETV), a neglected and re-emerging mosquito-borne alphavirus, has become more serious and poses a potential threat to animal safety and public health. Given the lack of antivirals and vaccines against GETV, further development of tools, including reverse genetics techniques, is crucial for combating this pathogen. Herein, we describe the design and construction of a DNA-launched infectious clone for GETV. The full-length genome of the GETV HuN1 strain, flanked by the cytomegalovirus immediate-early (CMV) promoter sequence at the 5' end and the hepatitis delta virus ribozyme along with the bovine growth hormone termination and polyadenylation signal sequences at the 3' end, was packaged in a bacterial artificial chromosome vector to establish the GETV infectious clone pBR322-GETV-HuN1. In parallel, recombinant reporter viruses carrying the reporter gene EGFP between the E1 gene and the 3' UTR were constructed on the basis of the established CMV-driven cDNA clone. Both in vivo and in vitro experiments have shown that the rescued recombinant virus (rGETV-HuN1 and rGETV-EGFP) possesses viral biological activity similar to that of the parental virus. In summary, this study develops a concise and efficient GETV infectious cDNA clone and a recombinant virus carrying an EGFP reporter gene. The availability of GETV infectious clones will facilitate further studies on understanding the molecular mechanisms of GETV biology, virulence determinants, molecular pathogenesis, vaccine development and virus‒host interactions.

## Introduction

Alphaviruses transmitted by arthropods are rapidly emerging and re-emerging human pathogens and continue to pose a global threat. Alphaviruses cause serious diseases in animals and humans, especially current epidemic strains, and increased virulence is caused by genetic mutations. Getah virus (GETV) is a member of the genus *Alphavirus* in the family *Togaviridae* and is an arthropod-borne virus. It was first isolated from naturally infected mosquitoes in Cambodia in 1966 [1]. Currently, Getah virus is distributed worldwide, with reports of virus isolation and animal outbreaks from regions such as Australia, Malaysia, Thailand, China, Japan, and Sri Lanka [2–7]. In recent years, GETV infections have been reported successively across various regions of China [8–11], causing significant losses to the livestock industry. As a member of the genus *Alphavirus*, Getah virus has a broad host range and is capable of infecting horses, pigs, foxes, and cattle [7, 12–14]. The clinical features of Getah virus-infected horses include depression, anorexia, fever, limb swelling, and lymphocyte reduction [13, 15, 16]. Additionally, Getah virus infection in pigs can lead to reproductive disorders in sows, such as mummified or stillborn piglets, as well as diarrhea and death in piglets [17, 18]. The lack of vaccines or antiviral measures is a major obstacle to the treatment of future epidemics. It is important to monitor GETV evolution carefully to prevent further public health issues.

Like typical alphaviruses, GETV has a typical alphavirus structure: a single-stranded RNA genome at the center of the viral particle surrounded by a capsid protein and enveloped by a lipid membrane and an outer glycoprotein shell composed of the E1 and E2 glycoproteins [19–24]. The genome is approximately 11.7 kb in length, with a 5' methylated cap structure and a 3' poly(A) tail. The genome contains two open reading frames (ORFs), which encode four nonstructural proteins (nsP1, nsP2, nsP3, and nsP4) and five structural proteins (Cap, E3, E2, 6 K, and E1). The GETV particles are spherical, approximately 70 nm in diameter, and are enveloped with spikes on the surface [25].

The increasing emergence of GETV poses a serious threat to animal health and public health. However, the etiology and viral determinants of GETV pathogenesis are limited. Reverse genetics systems provide powerful tools for the research of RNA viruses. It has been used to decipher the biological properties of viruses, and infectious full-length cDNA clones have been established for several RNA viruses [26]. Bacterial artificial chromosomes, which retain only one or two copies in a bacterium and enable the stability of 300 kb inserts, are used as backbones to increase the stability of infectious cDNA clones [27]. pBR322 is an important artificial plasmid known as a universal vector that is constructed through a complex recombination process involving three parental plasmids: pSF2124, pMB1, and pSC101 [28]. It is commonly used to achieve virus rescue by either transfecting in vitro-transcribed RNA or transfecting cDNA constructs into susceptible cells, and a chimeric intron accompanied by hammerhead ribozyme (HamRz) sequences is introduced at the beginning of the viral genome to increase the stability of the infectious clone. At present, Alphavirus species such as Chikungunya virus [29], Sindbis virus [30], Mayaro virus [31], and Venezuelan equine encephalitis virus [32] have successfully utilized reverse genetics to establish infectious full-length cDNA clones. In this study, we report the establishment of a full-length infectious cDNA clone on the basis of the isolated GETV HuN1 strain. Using the established CMV-driven cDNA clone, an EGFP recombinant reporter virus was also constructed for high-throughput drug screening in the future. This potential of DNA-launched GETV will be useful for establishing the molecular basis of replication, assembly, and pathogenesis, evaluating potential antiviral drugs, and as a vaccine platform for the development of DNA-based recombinant GETV vaccines.

## Materials and methods

### Cells, viruses, plasmids and reagents

The pBR322 plasmid (D2301) and trypsin were purchased from Beyotime Biotechnology; porcine-derived Getah virus strain HeN202009-2 (MZ736801.1) was isolated, identified, and stored at Anhui Animal Disease Prevention and Control Center; *E. coli* DH5α receptor cells, FastPfu DNA Polymerase, PBS and DMEM were purchased from TransGen Biotech; foetal bovine serum was purchased from ExCell Bio; restriction endonucleases *Xho* I, *Eco*R I, *Bgl* II and *Hin*d III were purchased from Takara; TIANamp Virus DNA/RNA Kit, Endotoxin-free Plasmid Extraction Kit were purchased from TIANGEN; Hifair V Reverse Transcriptase was purchased from Yeasen; Lipofectamine 3000 was purchased from Thermo Fisher; 2.5% glutaraldehyde electron microscope fixative was purchased from Solarbio; recombinant plasmids pCDNA3.1–3 × Flag and pEGFP-N1 vector, Baby Hamster Kidney 21 (BHK-21) and GETV E2 protein polyclonal antibodies were prepared and stored in our laboratory.

### Strategy for constructing the full-length cDNA cloning plasmid

**Figure 1:**
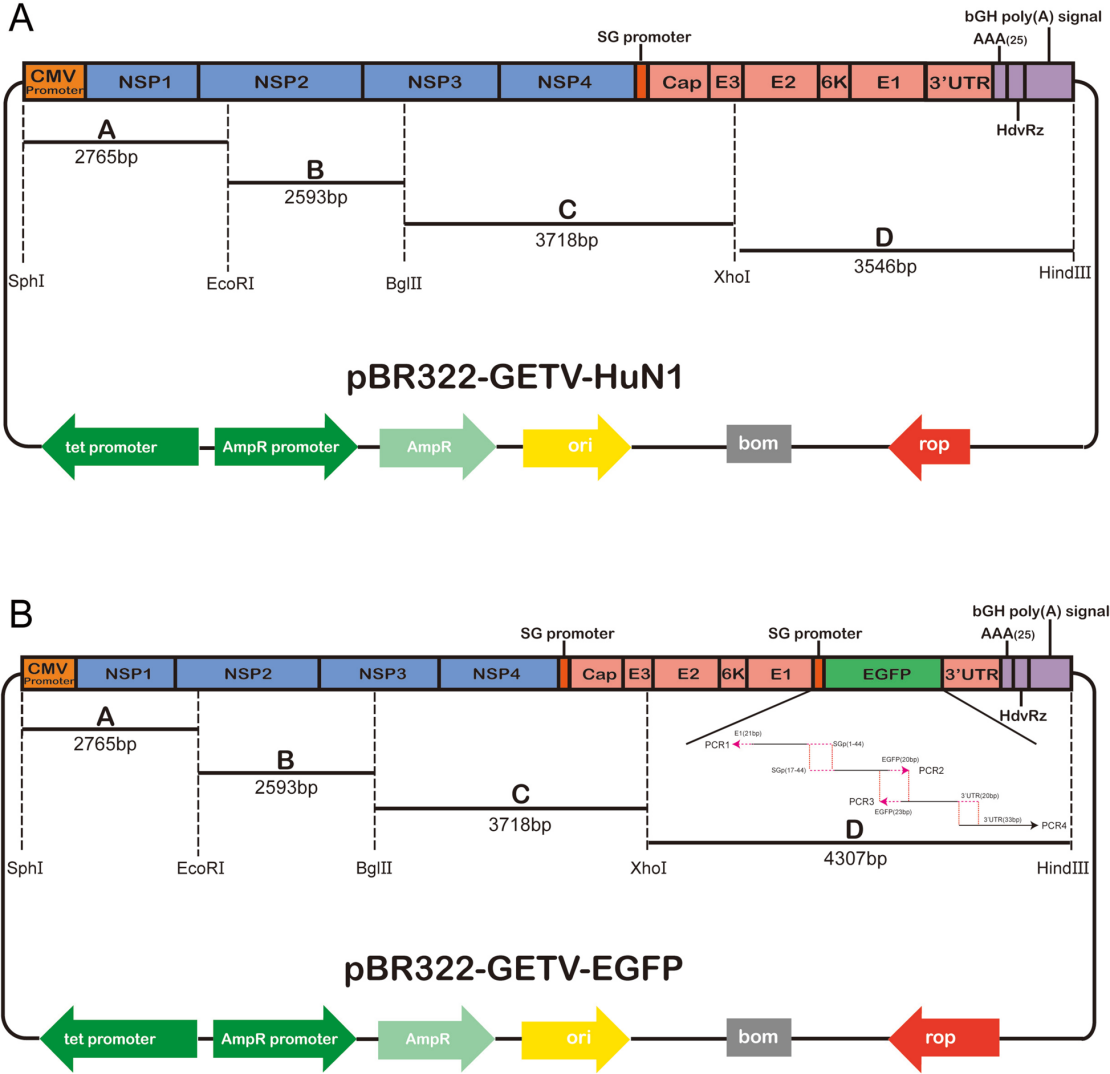
**Strategy for constructing full-length cDNA infectious clones of the GETV HuN1 strain and the strain carrying a genetic marker**. (**A**) The complete genome of the GETV HuN1 strain was divided into four fragments (A, B, C, and D) and cloned into the pBR322 vector. (**B**) The genome of the GETV HuN1 strain was partitioned into fragments A, B, C, and D, with fragment D containing an SG promoter and an EGFP tag inserted between the E1 coding region and the 3’UTR prior to cloning into the pBR322 vector

The genome sequence of the GETV HuN1 strain virus was divided into 8 segments for protein expression. The CMV promoter and bGH poly(A) tail nucleotide sequences were subsequently cloned from the pCDNA3.1( +) plasmid. These sequences were then inserted into the 5'UTR and 3'UTR of the GETV gene sequence using overlap PCR. The sequence of the hepatitis delta virus ribozyme was synthesized and then connected to the 3'UTR end of the GETV gene sequence via overlap PCR. The recombinant segments containing the CMV promoter, bGH poly(A) tail, and HdvRz were divided into four long segments labelled A, B, C, and D via overlap PCR or homologous recombination. These segments were sequentially cloned and inserted into the pBR322 vector to obtain a recombinant plasmid containing the full-length cDNA clone of the GETV genome, named pBR322-GETV-HuN1. The construction strategy is shown in Figure 1A. In the GETV infectious clone, a 44 bp subgenomic promoter was inserted at the 11 288 nt position of the viral genome as a molecular marker for the rescued virus. An enhanced green fluorescent protein (EGFP) sequence was inserted downstream of the molecular marker in the rescued virus as a genetic marker. The resulting recombinant plasmid, containing the correct full-length GETV genome cDNA clone, was named pBR322-GETV-EGFP. The construction strategy is shown in Figure 1B.

### Virus rescue

Two infectious clone plasmids were transfected into BHK-21 cells using Lipo3000, and the empty pBR322 plasmid was transfected as a negative control. After incubation at 37 °C for 2 h, the medium was discarded, and the medium was replaced with 5% fresh maintenance medium. The cells were then cultured at 37 °C in a 5% CO_2_ incubator. When cytopathic effects appeared in some cells, the cells and supernatants were collected for freeze‒thaw cycles. After centrifugation, the supernatant was harvested and inoculated into fresh BHK-21 cells. Following 10 blind passages, identification was performed. The supernatant of the modified GETV carrying the green fluorescent reporter gene after ten blind passages was collected. RNA was extracted, reverse transcribed into cDNA, and subjected to PCR amplification. The PCR products were gel-extracted and sent for sequencing.

### Detection of the rescued virus particles by electron microscopy

The cloned viruses rGETV-HuN1 and rGETV-EGFP were used to infect BHK-21 cell cultures. After centrifugation at 12 000 rpm (4 °C) for 30 min, 20 μL of the supernatant was adsorbed onto a carbon-coated copper grid for 5 min, stained with 2% phosphotungstic acid (pH 6.5) for 1 min, dried, and observed under a transmission electron microscope.

### Detection of rescued virus particles by an immunological fluorescence assay

BHK-21 cells were seeded into a 12-well plate and incubated to 50% confluence. The cells were inoculated with either parental or rescued viruses at a multiplicity of infection (MOI) of 0.5 and incubated at 37 °C for 2 h. The culture medium was then removed and replaced with 5% fresh maintenance medium. When cytopathic effects appeared, the medium was discarded, and the cells were washed twice with PBS. The cells were fixed with 4% paraformaldehyde at room temperature for 30 min, followed by two washes with PBS. A mixture of 0.2% Triton X-100 and 4% BSA (1:1 ratio) was used to permeabilize and block the cells for 30 min, followed by two washes with PBS. The cells were incubated with a sheep anti-mouse secondary antibody labelled with Dylight 594 for 1 h at room temperature in the dark, followed by two washes with PBS. Finally, the nuclei were stained with DAPI for 10 min at room temperature and washed twice with PBS. The fluorescence was observed and imaged with a Leica confocal microscope.

### Plaque assay and one-step growth curve establishment

A tenfold serial dilution of the virus mixture was prepared by mixing 100 μL of virus with 900 μL of DMEM. Each dilution (100 μL) was used to infect BHK-21 cell monolayers. After a 2-h incubation at 37 °C, the cells were overlaid with 2 mL of DMEM containing 5% FBS and 1% low-melting agarose. The cells were cultured at 37 °C in a 5% CO_2_ incubator for 4 days. The medium was removed, and the cells were fixed with 4% paraformaldehyde for 30 min, stained with 1% neutral red staining solution for 12 h, air dried naturally and counted for plaque numbers. BHK-21 cells were seeded in 6 cm cell culture dishes and allowed to reach 80% confluence. The cells were then infected separately with GETV-HeN, rGETV-HuN1 and rGETV-EGFP. Virus-containing supernatants were collected at 4, 8, 12, 16, 20, and 24 h postinfection, and viral titres (TCID_50_) were determined. Growth curves were plotted on the basis of the results.

### Genetic stability analysis of rescued Getah viruses

To test the genetic stability of the recombinant viruses, the rescued viruses were continuously passaged for 10 generations (P1–P10). The protein expression of Cap in GETV-infected cells at passages P1, P3, P5 and P9 was analysed by western blotting. EGFP expression in the green fluorescent reporter gene-modified GETVs at P1, P3, P5, P7, and P9 was monitored via laser confocal microscopy.

### Analysis of the pathogenicity of the rescued Getah virus in mice

The animal experiments were approved by the Institutional Animal Care and Use Committee (IACUC) of Anhui Agricultural University. The animals used in the experiments conformed to the ARRIVE guidelines for the welfare and ethics of laboratory animals at Anhui Agricultural University (AHAUB2023100). Three-day-old ICR suckling mice were selected, and the control and experimental groups were intracranially inoculated with DMEM and rGETV-HuN1, respectively. After inoculation, the condition of the suckling mice was monitored daily at fixed intervals, and body weight was recorded regularly. Suckling mice were necropsied after 84 h of infection. Deaths during the infection period were recorded, and the samples were necropsied immediately. After dissection, organs from infected and control mice were collected in EP tubes. RNA was extracted from various tissues of suckling mice and reverse transcribed into cDNA, and the viral load in the tissues was determined by quantitative PCR (qPCR).

### Histopathological examination of the mice infected with rescued Getah viruses

Pathological sections were prepared from tissues with significant clinical lesions and high viral loads. These tissues included heart, lung, brain and intestine. The tissues were fixed in 4% paraformaldehyde and dehydrated through a graded ethanol series. The dehydrated tissue samples were processed for paraffin embedding in paraffin blocks, sectioned, deparaffinized and stained with haematoxylin‒eosin (HE).

### Statistical analysis

The data are presented as the mean ± standard error of the mean (SEM) (standard deviation [SD]). Statistical analyses were performed with GraphPad Prism 8 software using two-way ANOVA followed by the Bonferroni post hoc correction or unpaired Student’s t test. Statistically significant and highly significant results were defined as *P* < 0.05 and *P* < 0.01.

## Results

### Construction of a full-length infectious clone of Getah virus and a clone with a genetic marker

**Figure 2:**
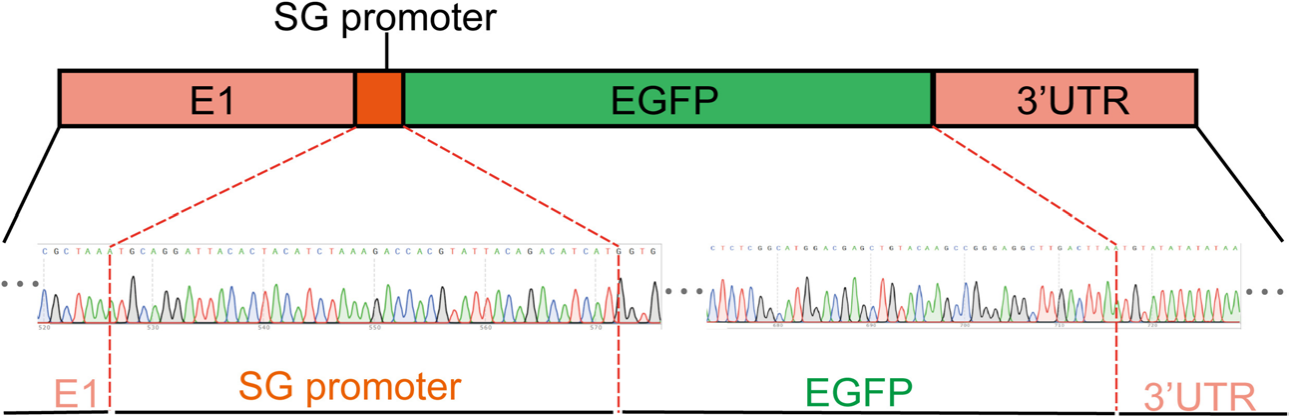
**Identification of the SG promoter and EGFP genes in the rescued virus.** The PCR fragment containing the subgenomic promoter was amplified by RT‒PCR from RNA extracted from the recombinant virus. The PCR products were then sequenced.

To facilitate the study of GETV HuN1, the entire genome of the GETV HuN1 strain was divided into eight fragments and cloned and inserted into the pBR322 vector, as described in the Methods section and shown in Figure 1A. The DNA-launched infectious clone contained a CMV promoter at the 5' end of the viral genome, the full-length genome sequence of the GETV HuN1 strain, a 25-nucleotide poly(A) tail at the 3' end of the genome, followed by HdvRz and bGH signal sequences. The strategy for constructing the EGFP-containing infection construct was cloning, as shown in Figure 1B. Sequence analysis confirmed that both clones retained full identity to the parental GETV HuN1 strain, with no base mutations detected. Sequencing confirmed successful insertion of the SG promoter and EGFP between the E1 and 3'UTR sequences (Figure 2). The sequences of the PCR primers used for each fragment are shown in Table 1.

**Table 1:** **Sequence of the primers**

Gene	Sequence (5′ → 3′)
cmv-(BamHI)-F(pcDNA3.1( +)3 × FLAG)	F:AGAGAATTCGATATCGGATCCGACATTGATTATTGACTAGTT
nsp2-(xhoI)-R(pcDNA3.1( +)3 × FLAG)	R:TTTAAACTTACCGGTCTCGAGGAATTCGTGGAAAGGTGGATTG
(NSP1-)nsp2-F	F:GAATTAACCTTCAGAGCCGGAGCCGGGGTTGTGGAAACACCCAG
NSP1(-nsp2)-R	R:CTGGGTGTTTCCACAACCCCGGCTCCGGCTCTGAAGGTTAATTC
(pcDNA3.1–3 × FLAG-)NSP2-F	F:AGAGAATTCGATATCGGATCCGAATTCGCATACGAAGGACTCAAGATC
(pcDNA3.1–3 × FLAG-)NSP3-R	R:TTTAAACTTACCGGTCTCGAGAGATCTGCTCGTTAGCATCTTGG
(pCAGGS-HA-EcoRI) BglI-NSP3-F	F:TTCCAGATTACGCTGAATTCAGATCTGCCTGTACGCCCTAG
SGp-C-E3(XhoI)-R(EcoRI-pCAGGS-HA)	R:GGTACCATCGATGAGCTCGAATTCCTCGAGCAGATCGTAGTAGCCC
(pCAGGS-HA-EcoRI-)E3-F	F:TTCCAGATTACGCTGAATTCCTCGAGGCCACGATGACGT
E3-3'UTR(-25A-HdvRz)-R	R:CTTCGGATGCCCAGGTCGGACCGCGAGGAGGTGGAGATGCCATGCCGACCCTTTTTTTTTTTTTTTTTTTTTTTTTGTAAAATATTAAAAAAACAAATTAG
(HdvRz-)BGH-F	F:CCGACCTGGGCATCCGAAGGAGGACGTCGTCCACTCGGATGGCTAAGGGAGAGGATCCCTGTGCCTTCTAGTTGCCAG
BGH(-pCAGGS-HA)-R	R:ACCATCGATGAGCTCGAATTCCCATAGAGCCCACCGCATCCCCA
(E1-SGp-)EGFP-F	F:CATCTAAAGACCACGTATTACAGACATCATGGTGAGCAAGGGCGAGGA
E1(-SGpromoter)-R	R:GTCTGTAATACGTGGTCTTTAGATGTAGTGTAATCCTGCATTTAGCGGCGCATAGTCACACACGT
(EGFP-)3'UTR-F	F:CCGGGAGGCTTGACTTAATGTATATATATAAGC
EGFP(-3'UTR)-R	R:CATTAAGTCAAGCCTCCCGGCTTGTACAGCTCGTCCATGCCGA
B-F	F:GACAGCTTATCATCGATAAGCTTGAATTCGCATACGAAGGACTCAAGAT
B-R	R:ATGCGTCCGGCGTAGAGGATCCCTCGAGAGATCTGCTCGTTAGCATCTTGG
C-F	F:TTCCAGATTACGCTGAATTCAGATCTGCCTGTACGCCCTAG
C-R	R:GGTACCATCGATGAGCTCGAATTCCTCGAGCAGATCGTAGTAGCCC
A-F	F:TGACAGCTTATCATCGATAAGCTTGAATTCGACATTGATTATTGA
A-R	R:GGATCTTGAGTCCTTCGTATGCGAATTCGTGGAAAGGTGGATTGATTA
D-F	F:GTGGACCGCCCGGGCTACTACGATCTGCTCGAGGCCACGATGACGTGTAACAAT
D-R	R:GCCACGATGCGTCCGGCGTAGAGGATCCCTCGAGCCATAGAGCCCACCGCATCC

### Significant cytopathic effects and observation of viral particles

**Figure 3:**
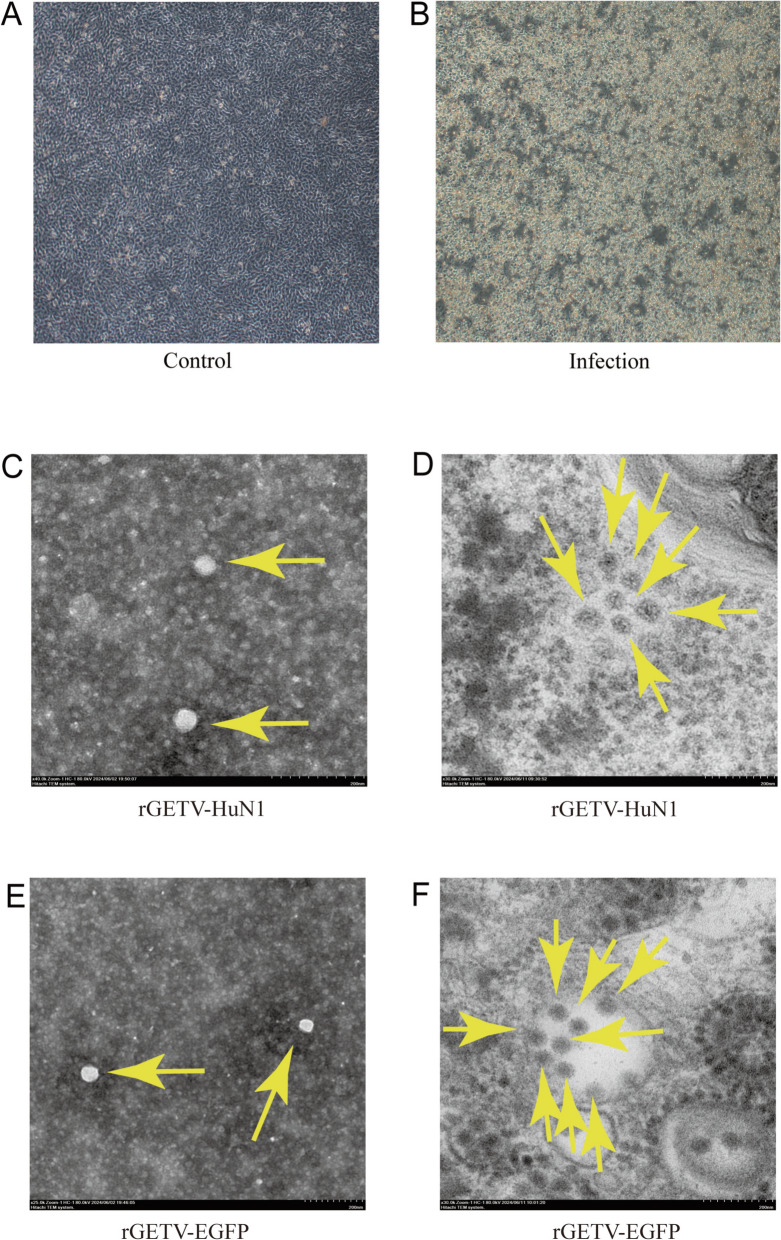
**CPE of GETV-infected BHK-21 cells and transmission electron microscopy (TEM) observations of rescued virus particles. A** BHK-21 cells in normal culture. **B** Cytopathic effects in BHK-21 cells infected with the cloned virus pBR322-GETV-HuN1. **C** Negative staining of the cloned virus pBR322-GETV-HuN1. **D** Ultrathin sections of the cloned virus pBR322-GETV-HuN1. **E** Negative staining of the cloned virus pBR322-GETV-EGFP. **F** Ultrathin sections of the cloned virus pBR322-GETV-EGFP.

The recombinant virus derived from the transfected pBR322-GETV-HuN1 plasmid exhibited distinct cytopathic effects (CPE) as early as the second passage, including cell rounding, shrinkage, fusion, and detachment, which were progressively enhanced in subsequent passages. As the rescued virus propagated through serial passages, CPE onset accelerated and became more pronounced, whereas control group cells retained a normal morphology with clear boundaries (Figures 3A and B). Negative-staining transmission electron microscopy revealed rescued viral particles (rGETV-HuN1 and rGETV-EGFP) with an average diameter of ~70 nm (Figures 3C and E). Ultrathin sections of infected BHK-21 cells confirmed the presence of both viral particle types by TEM (Figures 3D and F). These results demonstrate that the reverse genetics-derived infectious clones induced CPE and presented biological structures comparable to those of the wild-type virus.

### Generation and characterization of the recombinant virus

**Figure 4:**
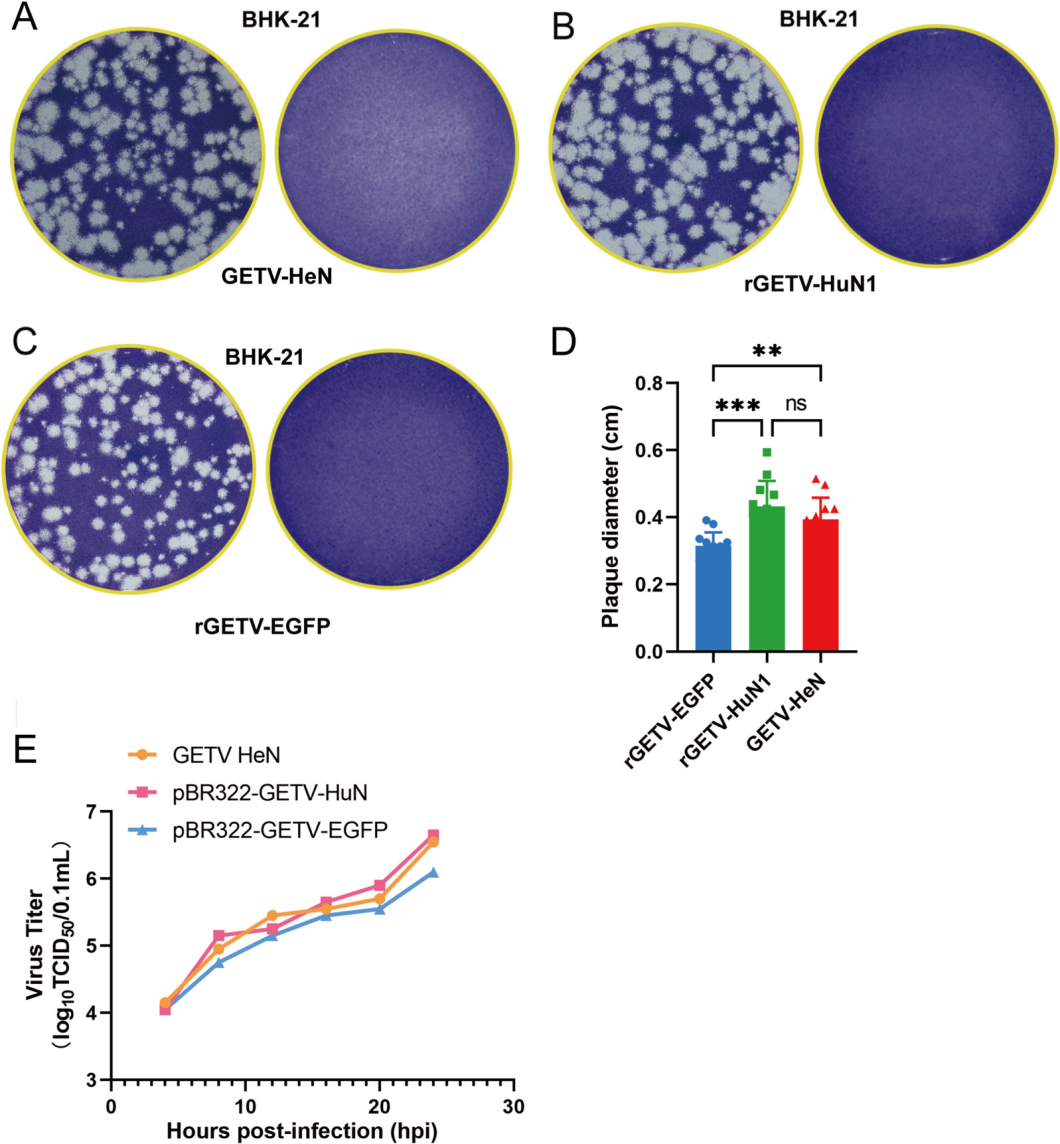
**Establishment of the plaque assay and one-step growth curve. A** Plaque morphology of GETV-HeN in BHK-21 cells stained with neutral red at 48 h. **B** Plaque morphology of rGETV-HuN1 in BHK-21 cells stained with crystal violet at 96 h. **C** Plaque morphology of rGETV-EGFP in BHK-21 cells stained with crystal violet at 96 h. **D** Statistical analysis of the diameter of empty spots (** *P* < 0.01, *** *P* < 0.001).** E** Growth curves of rescued and parental viruses in BHK-21 cells at an MOI of 0.5. Viral titres (TClD_50_) represent the means of three independent experiments.

To assess the biological activity of the rescued viruses, plaque assays and one-step growth curve analyses were conducted. To quantitatively assess phenotypic differences, we performed comparative plaque analysis across the rGETV-EGFP, rGETV-HuN1, and GETV-HeN strains. Twelve randomly selected plaques per experimental group were subjected to morphometric quantification, with plaque diameters measured in centimetres (cm) via standardized imaging protocols. One-way ANOVA with post hoc analysis demonstrated significant intergroup differences. Specifically, rGETV-HuN1 presented the largest mean plaque diameter (0.432 ± 0.076 cm), which surpassed those of both rGETV-EGFP (0.3229 ± 0.041 cm; *P* < 0.001) and GETV-HeN (0.394 ± 0.064 cm; *P* < 0.01). Quantitative comparisons revealed a 25.3% mean diameter reduction in rGETV-EGFP relative to rGETV-HuN1 (95% CI: 21.8–28.8%) and an 18.05% reduction compared to GETV-HeN (95% CI: 14.2–21.9%) (Figure 4D). For growth curve analysis, BHK-21 cells were infected with rGETV-HuN1, rGETV-EGFP, or the parental virus GETV-HeN at an MOI of 0.5. The supernatants collected at the indicated time points were used to determine the TCID_50_ values, and growth curves revealed that the rescued viruses presented replication kinetics and growth patterns similar to those of the parental virus (Figure 4E). These results confirm that the rescued Getah viruses retain biological activity comparable to that of the wild-type virus.

### Detection of rescued Getah virus and gene-marked rescued Getah virus via IFA

**Figure 5:**
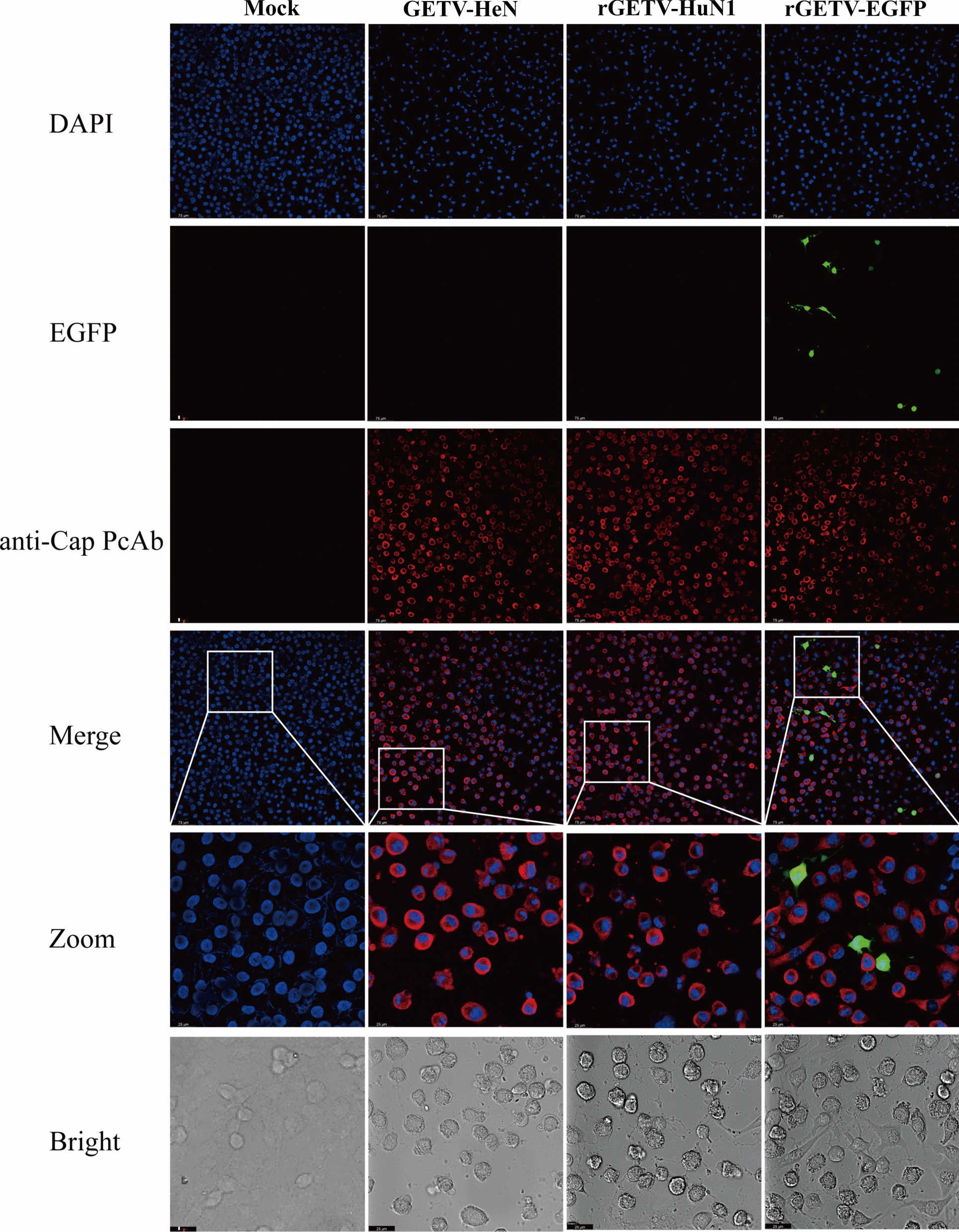
**IFA analysis of Cap protein expression in BHK-21 cells. A** BHK-21 cells infected with the parental virus pBR322-GETV-HuN1 or pBR322-GETV-EGFP were fixed and stained with anti-GETV-Cap PcAb and a goat anti-mouse secondary antibody labelled with Dylight594. Scale bar = 50 μm.

To confirm the presence of the virus and quantify rescued viruses, BHK-21 cells were infected with the parental virus (GETV-HeN) and two rescued viruses (rGETV-HuN1 and rGETV-EGFP). An immunofluorescence assay (IFA) using previously generated polyclonal antibodies against the GETV Cap protein demonstrated specific reactivity in infected cells, with distinct fluorescent signals observed in both the parental and rescued virus groups, whereas the negative controls showed no fluorescence (Figure 5). Bright-field imaging revealed nuclear shrinkage, fragmentation, and loss of intact cellular morphology in virus-infected cells, which contrasted sharply with the findings in control cells. These IFA results confirmed that the reverse genetics-derived Getah virus-infected clones were successfully rescued.

### Rescued Getah viruses can be genetically stabilized and replicated

**Figure 6:**
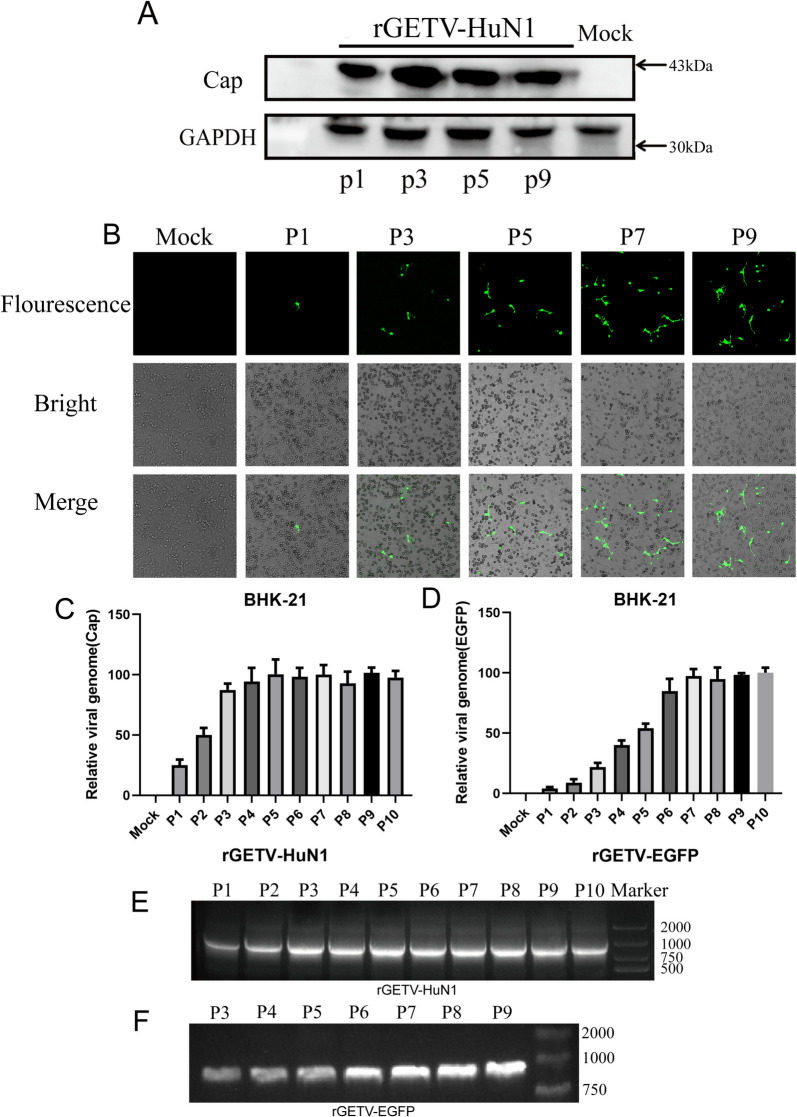
**Genetic stability of rescued Getah viruses**.** A** BHK-21 cells were infected with the rescued virus rGETV-HuN1 with first-generation, third-generation, fifth-generation, and ninth-generation viruses at an MOI of 1 for 24 h. **B** Expression of reporter autofluorescence. BHK-21 cells were seeded in 35 mm confocal dishes and infected with reporter viruses. At 24 h, live cells were imaged with a Leica laser confocal microscope. **C-D** Effects of rGETV-EGFP and rGETV-HuN1 on mRNA levels in ten different generations (P1 to P10). BHK-21 cells were infected with pBR322-GETV-EGFP or pBR322-GETV-HuN1. After 20 h, the viral supernatants were collected for RNA extraction, which was then reverse-transcribed into cDNA. The levels of *Cap* and *EGFP* RNA at 10 passages were quantified via qPCR. The results are presented as the mean ± SD. **E–F** Agarose gel images showing the specific DNA fragment generated by RT‒PCR from RNA extracted from BHK-21 cells infected with GETV infectious clones.

To determine whether the rescued Getah virus could sustain infection, we assessed viral activity via standard molecular biology techniques. Western blot analysis confirmed stable Cap protein expression during viral replication (Figure 6A). Laser confocal microscopy revealed progressive increases in EGFP-expressing virions with successive passages (Figure 6B), indicating consistent genetic characteristics of the rescued viruses. RT‒qPCR analysis showed initial upregulation followed by stabilization of both Cap expression in rGETV-HuN1 and EGFP expression in rGETV-EGFP (Figures 6C and D). The PCR band intensity patterns corroborated these findings (Figures 6E and D). These results collectively demonstrate that the rescued virus has high infectivity and sustains stable EGFP expression.

### Pathogenicity of the rescued Getah viruses in mice

**Figure 7:**
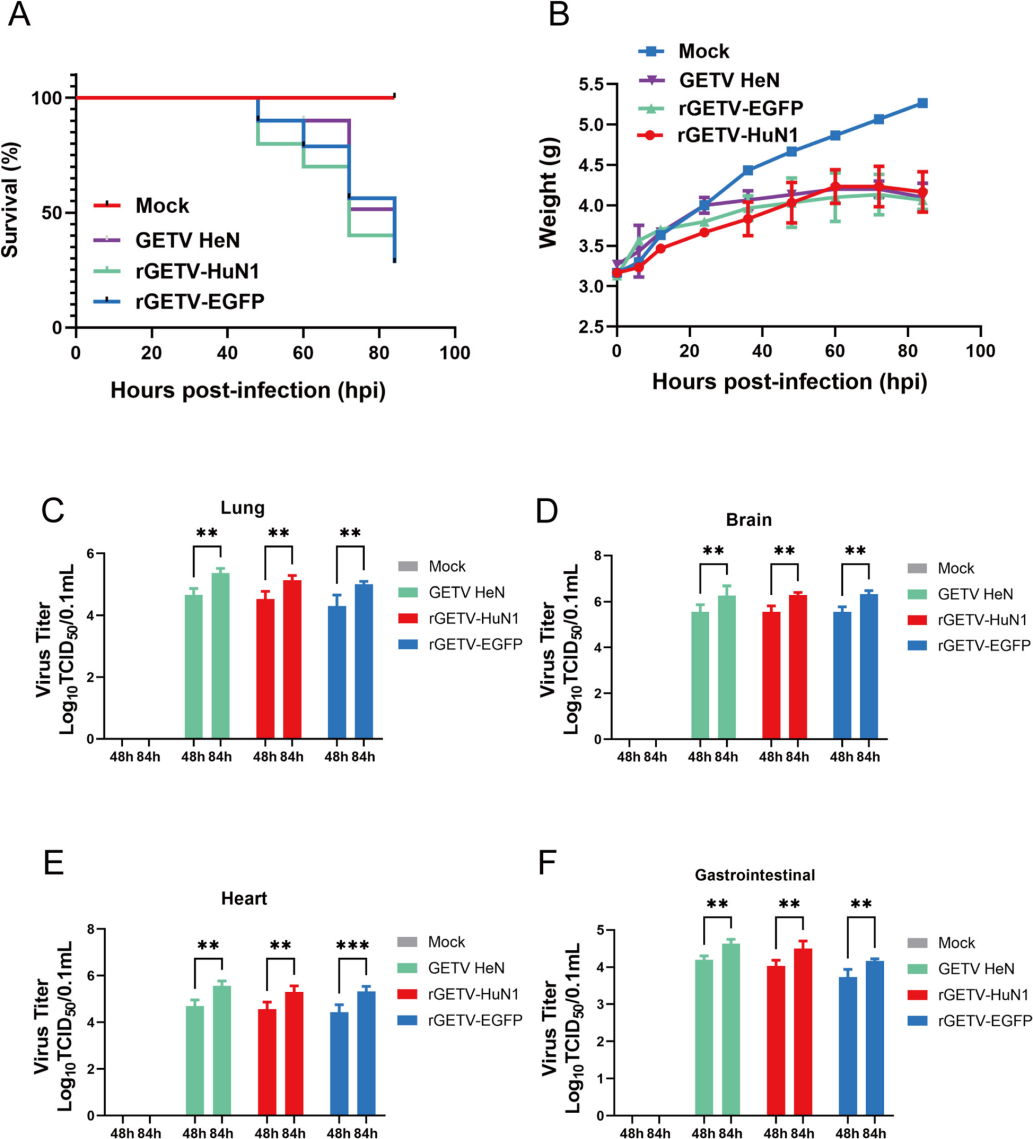
**Pathogenicity of the rescued viruses in mice. A** Survival curves of mice inoculated with two types of rescued virus and the parental virus. The data of the control group were recorded. All 10 mice in the control group survived and remained healthy. **B** Changes in the weights of the mice inoculated with GETV-infected clones and the control group were monitored (*P* < 0.001). **C**–**F**. Viral titres in the lung, brain, heart and gastrointestinal tract at 48 and 84 h postinfection. The mice were infected with mock (gray), GETV HeN (green), rGETV-HuN1 (red), or rGETV-EGFP (blue) vectors. The data are presented as log_10_TCID_50_/mL values (*n* = 3 per group).

To assess viral pathogenicity and in vivo distribution, 3-day-old ICR suckling mice received intracranial injections of either parental or rescued virus (1 MOI per mouse). The controls received an equivalent volume of DMEM. Within 84 h postinoculation, 70% mortality (7/10 mice) occurred in the infected groups, whereas the controls remained asymptomatic and grew normally (Figure 7A). The body weight at 84 h was significantly lower in the infected mice than in the control mice (*P* < 0.001), as shown in Figure 7B. Viral titres (TCID_50_) measured in heart, intestine, lung, and brain tissues at 48/84 hpi revealed brain-specific viral accumulation. Notably, the brain titres at 84 hpi exceeded those at 48 hpi (Figures 7C–F).

### Pathological evaluation of organ lesions and tissue changes in mice infected with the virus

**Figure 8:**
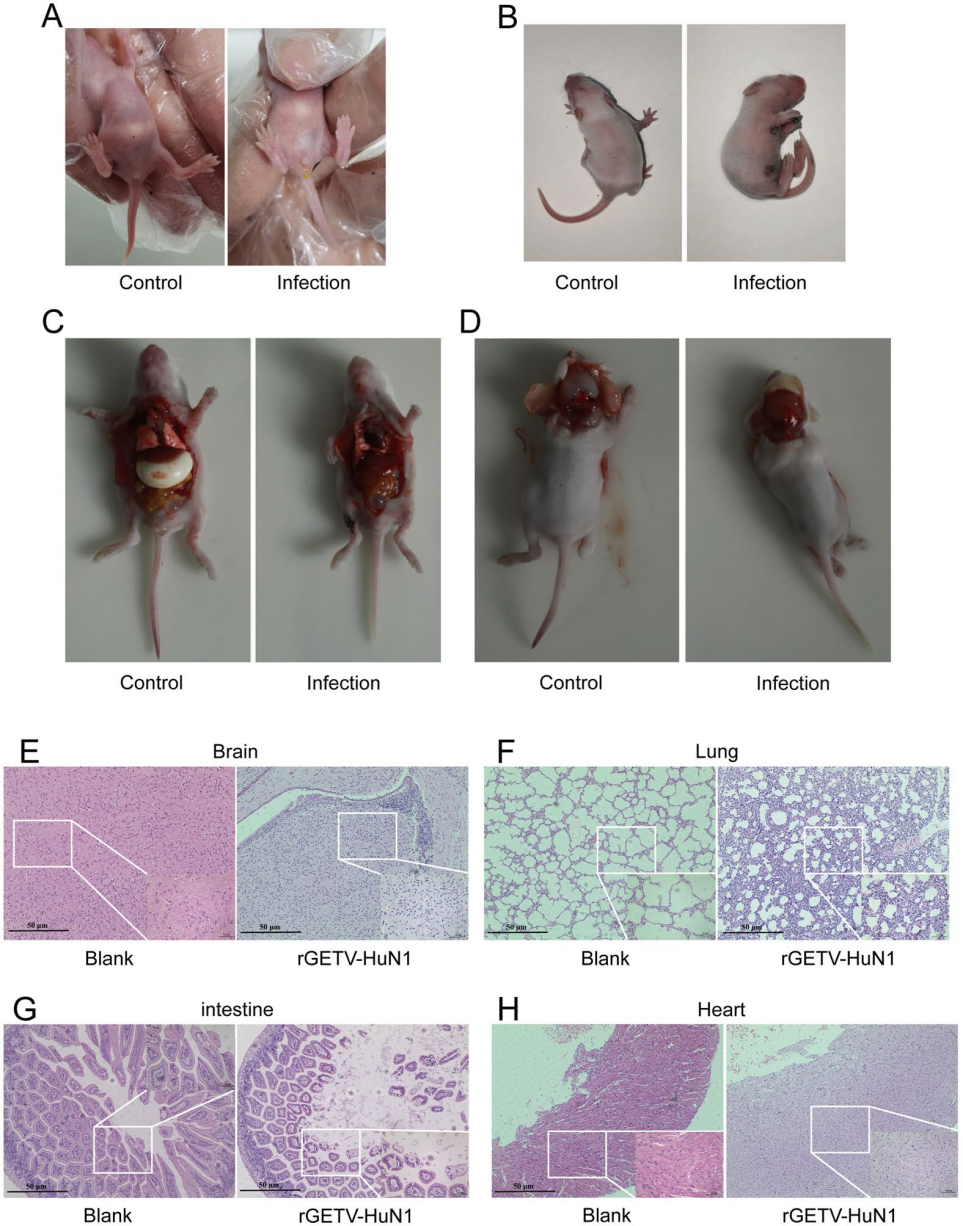
**Pathological section results of GETV infectious clones**.** A** Seventy-two hours after infection with the rescued virus rGETV-HuN1, the perianal region was compared between the control group and the infected group. **B** After 72 h of infection with the recombinant virus, the morphology of the control group and the infected group was compared. **C** Eighty-four hours after infection, the changes in the abdominal organs through dissection were compared between the control group and the infected group. **D** Eighty-four hours after infection, the changes in brain tissue were compared through dissection between the control group and the infected group. **E** Comparison of histopathological sections of brain tissue between the infected group and the control group. **F** Comparison of histopathological sections of lung tissue between the infected group and the control group. **G** Comparison of histopathological intestinal tissue sections between the infected group and the control group. **H** Comparison of histopathological sections of myocardial tissue between the infected group and the control group.

To evaluate the pathological effects of the rescued virus in mice, we conducted systematic behavioral and anatomical analyses. Within 24 h postinfection, the challenged mice developed anorexia, lethargy, perianal yellow water discharge, and neurological depression compared with the controls (Figure 8A). By 72 h, infected mice exhibited pronounced kyphosis and growth retardation (Figure 8B). Gross pathology revealed a reduced gastric volume with empty lumens in infected mice, whereas food-filled stomachs were reduced in controls (Figure 8C). Neuropathological examination revealed cerebral vascular congestion, perivascular inflammation, and microthrombi in infected mice, along with substantial inflammatory cell infiltration (Figures 8D and E). The lung tissues showed marked alveolar interstitial congestion and hyperplasia (Figure 8F), whereas the intestinal pathology revealed villous epithelial detachment and lactational diarrhea (Figure 8G). Cardiac histopathology revealed inflammatory infiltration, disorganized myofiber arrangement, and cardiomegaly (Figure 8H).

## Discussion

The establishment of a reverse genetics platform for GETV allows for the modification and manipulation of its genes at the DNA level. The DNA-launched infectious system is a useful tool with high rescue efficiency that allows the introduction of mutations in specific positions to investigate the function of an individual viral element. Rescued virus particles can be harvested by directly transfecting the DNA-launched recombinant plasmid into host cells, which reduces labor and experimental costs by eliminating the need for an in vitro transcription assay.

By observing phenotypic changes in rescued viruses, the functions of corresponding genes can be determined. This enables a deeper understanding of the structure and function of the GETV genome as well as its pathogenic mechanisms. Such insights are crucial for addressing the growing threat of GETV to livestock health, poultry farming, and public health safety in China. Previously, Ren et al. [33] successfully cloned the complete sequence of the GETV GX201808 strain into the pBR322 vector. They developed a reverse genetics system for the GETV GX201808 strain under the control of the 3' CMV promoter and the 5' bGH poly(A) signal sequence. Compared with the parental virus, the infectious clone of GETV cDNA contains seven nucleotide mutations, resulting in six amino acid changes. Although the growth kinetics are similar to those of the parental virus, the clinical symptoms, mortality rate, and brain tissue pathology of infected mice are indistinguishable from those caused by the parental virus. However, the time to death in mice infected with the infectious clone was delayed, which is likely due to the six nucleotide mutations in the cloned genome. Wang et al. [34] established a reverse genetics system for GETV by cloning the GETV-HN strain into the PSMART-LCKAN plasmid. This was achieved by introducing a CMV promoter in the 3'UTR and incorporating the HdvRz hepatitis delta virus ribozyme sequence and bGH poly(A) signal sequence in the 5'UTR. They successfully rescued the wild-type rGETV-HN and the mutant viruses E2-K253R and E2-K253A. Du et al. [35] introduced HamRz and HdvRz ribozyme sequences after the 3' UTR of the full-length GETV cDNA sequence, constructing a stable infectious cDNA clone named pGETV-SC483. Although the growth characteristics were similar to those of the parental virus, noticeable cytopathic effects (CPEs) were observed only two days post-transfection. This differs from the time to CPE appearance in the infectious clone constructed by Wang et al. [34].

In this study, two infectious clone plasmids, pBR322-GETV-HuN1 and pBR322-GETV-EGFP, were constructed. In the first and second generations of transfected BHK-21 cells, cytopathic effects (CPEs) were not easily observed. However, as the number of passages increased, the CPE became more apparent. This observation is different from the experimental results reported by Du et al. [35] and Ren et al. [33]. In the constructed pBR322-GETV-EGFP infectious clone plasmid, it was initially challenging to detect the EGFP reporter gene in the first two passages of the virus. With increasing passages, EGFP expression subsequently became more apparent. We hypothesize that the low proportion of EGFP-expressing GETV particles within the total rescued viral population may result from the construction strategy of the GETV infectious clone plasmid carrying the green fluorescent protein (EGFP) marker gene. Specifically, the retention of a stop codon following the E1 protein-encoding gene sequence, intended to preserve the integrity of the viral genome, might partially account for the weak EGFP expression observed in early passages. As the duration of infection increases, the viral population may have selectively enriched variants with increased EGFP expression due to improved adaptability, stability, or replication efficiency. It is possible that the initial viral population contained a mixture of viruses with varying levels of EGFP expression, and after multiple passages, viruses with stronger EGFP expression may have proliferated due to potential advantages such as enhanced promoter activity, better integration into the host genome, or improved expression of viral proteins. Additionally, epigenetic modifications or dynamic changes in the cellular environment during passaging could have contributed to this progressive increase in the EGFP signal.

Therefore, on the basis of previous studies, this research employed overlap PCR to add a CMV promoter upstream of the 5' UTR of the GETV genome and introduced a continuous sequence of 25 adenosine triphosphates (ATP), an HDVRz sequence, and a bGH poly(A) signal sequence downstream of the 3' UTR. This successfully established a more efficient and rapid reverse genetic system for the GETV HuN1 strain.

In this study, for the first time, the hepatitis delta virus ribozyme (HDVRz) sequence, the bovine growth hormone poly(A) tail sequence (bGH poly(A)) and the cytomegalovirus promoter (CMV) sequence were simultaneously inserted into both ends of the genome to construct a genetically stable GETV infectious cDNA clone. In addition, on the basis of the established infectious clone, we constructed a GETV infectious clone that stably expresses the EGFP reporter gene. The infectious clones constructed using this method could be successfully rescued through liposome transfection, resulting in the production of recombinant viruses that could be stably passaged. We believe that the establishment of the GETV reverse genetics system will facilitate further studies to understand the molecular mechanisms of GETV biology, virulence determinants, molecular pathogenesis, vaccine development and virus‒host interactions.

## Data Availability

The data generated during this study are available from the corresponding authors upon reasonable request.
